# Neglected brucellosis in pediatric populations from non-endemic regions: Clinical manifestations and prediction of severe disease in Yunnan Province, China

**DOI:** 10.1371/journal.pntd.0013645

**Published:** 2025-10-27

**Authors:** Xin Ma, Penghao Cui, Houyu Chen, Yan Guo, Yi Huang, Xiaotao Yang, Ying Zhu, Houxi Bai, Feng Jiao, Haifeng Jin, Ruonan Li, Qingping Tang, Yanchun Wang, Yonghan Luo

**Affiliations:** 1 Second Department of Infectious Disease, Kunming Children’s Hospital (Children’s Hospital Affiliated to Kunming Medical University), Kunming, Yunnan, China; 2 Faculty of Life Science and Technology, Kunming University of Science and Technology, Kunming, Yunnan, China; 3 Department of Reproductive Gynecology, NHC Key Laboratory of Healthy Birth and Birth Defect Prevention in Western China First People’s Hospital of Yunnan Province, Kunming, Yunnan, China; 4 Department of Reproductive Gynecology, The Affiliated Hospital of Kunming University of Science and Technology, Kunming, Yunnan, China; Colorado State University, UNITED STATES OF AMERICA

## Abstract

**Background:**

Although Yunnan Province is not an endemic region for brucellosis, the disease remains a diagnostic and therapeutic challenge in children due to its atypical clinical manifestations and potential for severe complications.

**Objective:**

This study aims to explore the clinical features of pediatric brucellosis in the region and establish a prediction model for severe complications.

**Methods:**

This study included 62 children diagnosed with brucellosis at the Kunming Children’s Hospital between 2015 and 2024. The patients were divided into two groups based on the presence of severe complications: the severe complications group (n = 15) and the general group (n = 47). Clinical features were extracted from electronic medical records, and the Boruta algorithm was used to select core predictive factors. Six machine learning models, including Random Forest and XGBoost, were constructed. The performance of the models was assessed using receiver operating characteristic curve (ROC) curves and decision curve analysis (DCA), and a web-based prediction tool was developed.

**Results:**

The study revealed that the most common clinical symptoms were fever (95.2%), joint pain (51.6%). Meningoencephalitis was observed in 13 cases (21%), and sacroiliitis was present in 2 cases (3%). Laboratory findings indicated that the erythrocyte sedimentation rate (ESR) and IgM levels were significantly higher in the severe complications group compared to the general group. Culture results showed that the positive rate of bone marrow cultures was 95% (19/20), blood cultures had a positive rate of 84% (52/62), synovial fluid cultures had a positive rate of 67% (2/3), and cerebrospinal fluid cultures had a low positive rate of 2% (1/43). Machine learning models demonstrated that the Random Forest model performed best in predicting severe complications (AUC = 0.970), and DCA indicated that it had the best clinical utility. Key predictive factors were disease duration, fever duration, IgM, and ESR. A Shiny-based web tool was developed for real-time clinical risk assessment.

**Conclusion:**

This study indicated that pediatric brucellosis should not be neglected in non-endemic areas like Yunnan Province, China. Combining inflammatory markers with Random Forest models can effectively predict the risk of severe complications in pediatric brucellosis.

## Author summary

Brucellosis is a neglected zoonotic disease that is frequently overlooked in non-endemic regions. We conducted a 10-year retrospective analysis of 62 pediatric cases of brucellosis in Yunnan Province, China, to investigate the clinical characteristics and risk factors for severe complications. The results revealed that fever and joint pain were the most common symptoms, while meningoencephalitis represented the predominant severe complication. Laboratory findings indicated significantly elevated erythrocyte sedimentation rate (ESR) and IgM levels in children with severe complications. Using the Boruta algorithm, we identified disease course, duration of fever, ESR, and IgM as key predictive factors. Among the six evaluated machine learning models, the random forest algorithm achieved the highest performance (AUC = 0.97) and was employed to develop a web-based calculator for real-time clinical risk assessment. Our findings underscore the need for heightened awareness of pediatric brucellosis in non-endemic regions and demonstrate that integrating inflammatory biomarkers with machine learning can effectively predict the risk of severe complications.

## 1. Introduction

Brucellosis is a globally distributed zoonotic disease [1]. In recent years, it has continued to be prevalent in Asia and Africa, with Asia bearing the highest burden, particularly in East, Central, and Western Asia, posing a significant public health challenge [2]. Despite its traditional association with livestock workers, sporadic cases in non-endemic areas have been increasing annually. Studies [2,3] indicated that the geographical spread of brucellosis has expanded significantly, from 53 countries to at least 97 countries.

In China, brucellosis is primarily endemic in regions with developed livestock industries, such as Inner Mongolia, Ningxia, Xinjiang, and Gansu, while Yunnan, a non-livestock region, reported an incidence rate of only 1.4806 per 10,000 population in 2021 [4]. Epidemiological data indicated that the disease predominantly affects adults, with a substantial number of cases arising from direct contact with infected animals or the consumption of unpasteurized dairy products [5]. Therefore, the pediatric population in Yunnan, a non-endemic area, is at greater risk of being neglected.

To our knowledge, there are no reports in the literature regarding the clinical and epidemiological characteristics of brucellosis in children in Yunnan Province. Typical symptoms of brucellosis in adult patients include recurrent fever, headache, migratory arthralgia, weakness, and myalgia [6]. Severe complications, such as meningitis, encephalitis, and infective endocarditis, can be fatal [7]. The epidemiological history of pediatric cases from non-endemic areas is often unclear, and the disease’s atypical clinical presentation and the lack of specific laboratory tests make it prone to misdiagnosis [8]. Furthermore, predictions regarding severe complications of brucellosis, such as neurobrucellosis and spondylitis, rely mostly on retrospective clinical experience and lack quantitative tools. Current research has predominantly focused on adult cases in endemic regions [6,9–12].

Given the above considerations, this study is the first to report on the clinical and epidemiological characteristics of brucellosis in children at a single-center pediatric hospital in Kunming, Yunnan Province. Additionally, a predictive model for severe complications in children was developed using machine learning, providing evidence-based support for optimizing clinical pathways.

## 2. Materials and methods

### 2.1. Study population

#### 2.1.1. Ethics statement.

A total of 62 pediatric patients diagnosed with brucellosis and receiving treatment at Kunming Children’s Hospital between 2015 and 2024 were included in the study. This study was approved by the Ethics Committee of Kunming Children’s Hospital and adhered to the principles outlined in the Declaration of Helsinki. Although informed consent was waived due to the retrospective nature of the study, this study was approved by the Ethics Review Committee of Kunming Children’s Hospital(2024-06-047-k05). Furthermore, we ensured that all patient data were anonymized to maintain confidentiality and privacy, in accordance with ethical standards. This study was carried out in accordance with the ethical standards of the Declaration of Helsinki.

### 2.2. Inclusion criteria and exclusion criteria

#### 2.2.1. Inclusion criteria.

a) Patients aged under 18 years.
b) Diagnosis of brucellosis based on clinical and confirmatory diagnostic criteria as per the “Diagnosis and Treatment Scheme for Brucellosis **(2023 Edition)**” [13], which include:
  I) Etiological Diagnosis: Isolation of Brucella from any pathological material such as blood, bone marrow, cerebrospinal fluid, pus, or other body fluids or excretions. In our study, all children underwent microbiological cultures, including blood culture, cerebrospinal fluid culture, and pus culture when clinically indicated. In suspected cases, repeated blood cultures were routinely performed, and the culture bottles used contained nutrient-rich media superior to standard broth, providing enhanced conditions for Brucella growth.
  II) Initial Screening Tests:(1) Positive result from the Rose Bengal Test (RBT). (2) Positive result from the Colloidal Gold Immunochromatographic Assay (GICA). (3) Positive result from Enzyme-Linked Immunosorbent Assay (ELISA, semi-quantitative, with manufacturer-recommended cut-off values for IgG and IgM antibodies. All ELISA tests were performed on the same platform, and numerical comparisons were made only within this system).
  III) Confirmatory Tests:(1) Tube Agglutination Test (SAT) with a titer of 1:100 or higher, or in cases with persistent clinical symptoms for more than one year, a titer of 1:50 or higher. (2) Complement Fixation Test (CFT) with a titer of 1:10 or higher. (3) Coombs Test with a titer of 1:400 or higher.

**Clinical Diagnosis:** Suspected cases with any positive result in initial screening tests. **Confirmed Diagnosis:** Cases with clinical or suspected diagnosis that are confirmed by any positive result in etiological diagnosis or serological confirmatory tests.

### 2.2.2. Exclusion criteria

a) Coexistence with other acute infectious pathogens.
b) Insufficient clinical information.
c) A history of bronchopulmonary dysplasia, congenital heart disease, hematologic malignancies, inherited metabolic disorders, primary immunodeficiency, or prior use of immunosuppressive drugs.

Based on the confirmed diagnosis of brucellosis, the occurrence of severe complications was assessed. Patients were divided into two groups according to the presence or absence of severe complications. These severe complications were defined as sacroiliitis, neurological involvement, and endocarditis. According to the “Diagnosis and Treatment Scheme for Brucellosis **(2023 Edition)**” central nervous system involvement and endocarditis are the primary causes of mortality in brucellosis patients. This classification is supported by treatment recommendations: uncomplicated brucellosis requires at least six weeks of therapy, whereas cases complicated with sacroiliitis, meningoencephalitis, or endocarditis require prolonged therapy of at least three months. The presence of these severe complications requires a more aggressive therapeutic approach, including a combination of three antimicrobial agents and extended treatment duration.

### 2.3. Study variables and data extraction

Clinical characteristic indicators were extracted from the hospital’s electronic medical record system, including General Information, Symptoms and Signs, Laboratory Tests, Treatment, Effect, and Outcomes.

### 2.4. Definition

Treatment success was characterized by the complete resolution of symptoms and signs of active disease by the end of therapy. Patients were considered to be in remission during follow-up if they remained asymptomatic with normal inflammatory markers.

Relapse was strictly defined as the reappearance of clinical symptoms consistent with active brucellosis, combined with either microbiological confirmation (a positive culture from blood or other sterile sites) or a significant serological rebound (a ≥ 4-fold increase in Brucella Sero agglutination titers) during the follow-up period of at least 6 months.

The diagnosis of meningoencephalitis was established by:(1) clinical symptoms such as persistent headache, altered consciousness, seizures, or focal neurological deficits;

(2) Cerebrospinal fluid(CSF) pleocytosis, meningeal enhancement, or parenchymal inflammation on contrast-enhanced brain computed tomography(CT) or MRI; and(3) exclusion of other common infectious or non-infectious causes.

### 2.5. Statistical methods

Data were analyzed using R statistical software (version 4.4.1). Continuous variables with a normal distribution were presented as mean ± standard deviation (x ± s), and comparisons between two groups were performed using independent t-tests. For non-normally distributed continuous variables, data were presented as median (interquartile range) [M (QR)], and comparisons between groups were conducted using the Mann-Whitney U test. Categorical variables were described using frequency and percentage [n (%)], and group differences were assessed using the chi-squared test (χ²) or Fisher’s exact test.

To establish a predictive model for severe complications, univariate predictive performance was first assessed using Receiver Operating Characteristic Curve (ROC curves). Although the overall sample size was modest (62 cases, with 15 in the severe complication group), a post-hoc power analysis indicated that this number of severe cases was sufficient to support ROC analysis with statistical significance. To enhance robustness, bootstrapping with 1,000 resamples was applied for internal validation. All analyses were conducted under the supervision of a professional biostatistician.The ‘Boruta’algorithm (1000 iterations) was employed to identify core predictive factors. Six machine learning models (logistic regression, K-Nearest Neighbors (KNN), naive Bayes, Multilayer Perceptron (MLP), XG-Boost, and random forest) were constructed, and model performance was evaluated using area under the curve (AUC), F1 score, accuracy, sensitivity, and specificity. Finally, clinical utility was validated using Decision Curve Analysis (DCA), and a prediction tool was developed using a web-based calculator based on ‘Shiny’. The significance level was set at α = 0.05.

## 3. Results

### 3.1. Basic information

**Fig 1:**
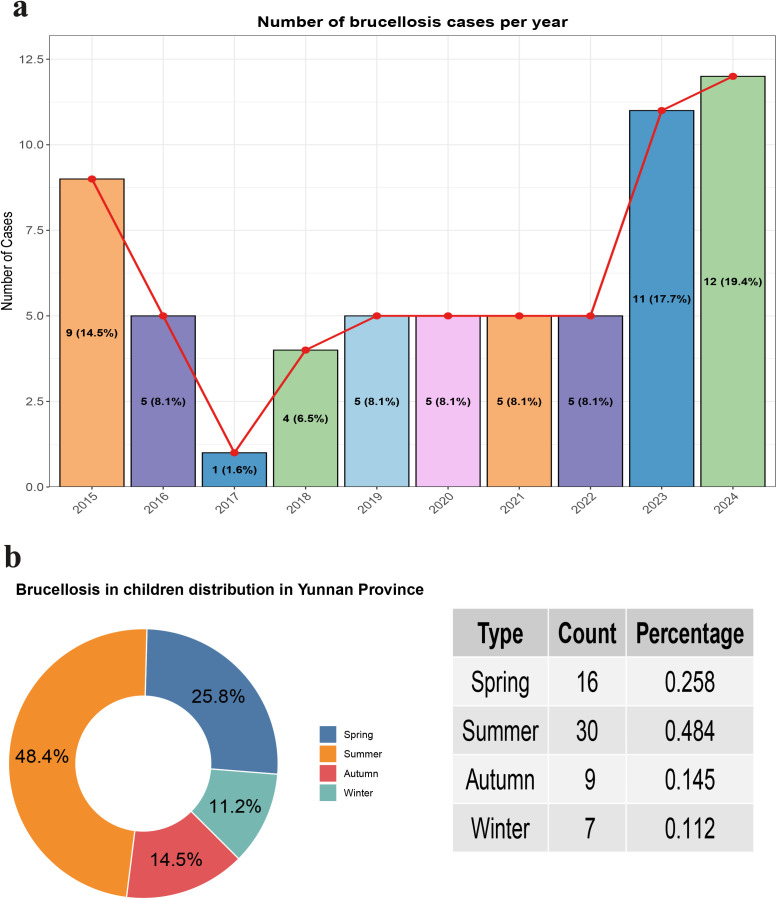
Incidence of pediatric brucellosis by year (a) and seasonal distribution (b) in Yunnan Province, China, 2015–2024.

This study compiled data on 62 confirmed cases of brucellosis in children between 2015 and 2024. The number of cases remained low and relatively stable in earlier years but showed a marked increase in 2023 (17.7%) and 2024 (19.4%), representing the highest incidence over the study period (**Fig 1a**).

Regarding seasonal distribution, summer was the peak season with 30 cases (48.4%), followed by spring with 16 cases (25.8%). The number of cases in autumn and winter was lower, with 9 cases (14.5%) and 7 cases (11.2%), respectively (**Fig 1b**).

**Fig 2:**
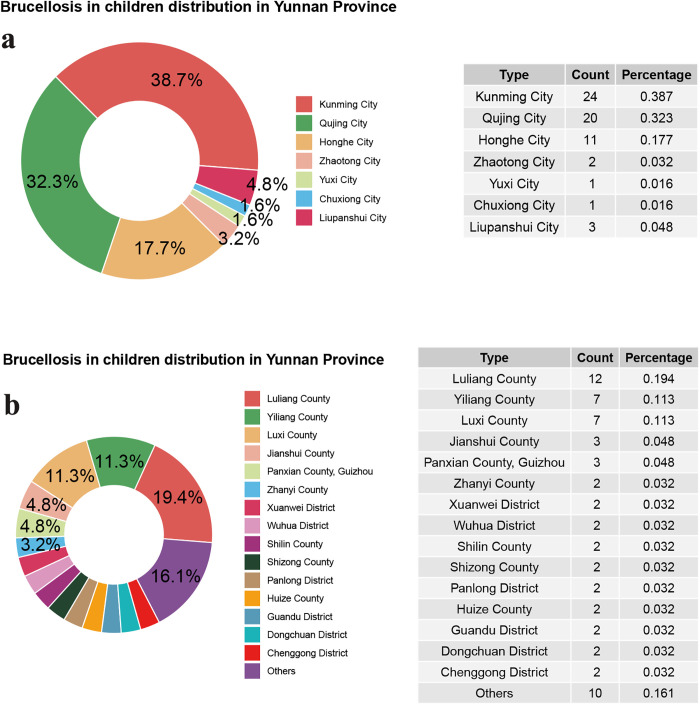
Geographic distribution of pediatric brucellosis in Yunnan Province, China, 2015–2024: a. distribution at the municipal level; b. distribution at the district level.

In terms of geographical distribution, Kunming City reported the highest number of cases, with 24 cases (38.7%), followed by Qujing City with 20 cases (32.3%), Honghe with 11 cases (17.7%), Zhaotong City with 2 cases (3.2%), Yuxi City and Chuxiong Prefecture each with 1 case (1.6%), and Liupanshui City with 3 cases (4.8%) (**Fig 2a**). At the county level, Luliang County reported the highest number, with 12 cases (19.4%), followed by Yiliang County and Luxi County each with 7 cases (11.3%). Other counties had fewer cases, with a total of 10 cases (16.1%) across various regions (**Fig 2b**).

Among the 62 children enrolled, 37 were male (60%) and 25 were female (40%). The median age of patients was 4.91 years, with the general group (n = 47) having a median age of 4.91 years and the severe complication group (n = 15) having a median age of 4.5 years. No statistically significant age difference was found between the groups (p = 0.622). The median weight was 15 kg for all patients, with the general group at 15 kg and the severe complication group at 17.5 kg, with no significant statistical difference (p = 0.791). The median disease duration was 25 days, with the general group at 24 days and the severe complication group at 34 days. Although there was a noticeable difference, it did not reach statistical significance (p = 0.118). Additionally, no significant differences were observed between the groups in terms of animal contact history and diagnosis time (**Table 1).**

**Table 1:** Demographic, clinical, and laboratory characteristics of pediatric brucellosis patients in Yunnan Province, China, 2015–2024.

Variables	Total (n = 62)	General group (n = 47)	Severe complication group (n = 15)	p-value
Basic Information				
Age (years), Median (Q1, Q3)	4.91 (2.35, 7.87)	4.91 (2.5, 7.91)	4.5 (2.24, 6.62)	0.622
Weight (kg), Median (Q1, Q3)	15 (12, 23)	15 (12, 23)	17.5 (12.25, 21.5)	0.791
Disease duration (days), Median (Q1, Q3)	25 (21, 36.75)	24 (21, 33.5)	34 (23, 40.5)	0.118
Male gender, n (%)	37 (60)	28 (60)	9 (60)	1
Rural residence, n (%)				1
Urban	7 (11)	6 (13)	1 (7)	
Rural	55 (89)	41 (87)	14 (93)	
Animal contact history, n (%)	60 (97)	45 (96)	15 (100)	1
Time to diagnosis (days), Median (Q1, Q3)	5 (5, 6)	5 (5, 7)	5.5 (5, 6)	0.811
Symptoms				
Fever, n (%)	59 (95)	44 (94)	15 (100)	1
Peak temperature (°C), Median (Q1, Q3)	39.5 (39, 40)	39.5 (39, 40)	39.9 (39.25, 40.1)	0.32
Fever duration (days), Median (Q1, Q3)	15 (7.75, 25.75)	15 (6.5, 19.5)	20 (12, 33)	0.073
Pre-hospital fever (days), Median (Q1, Q3)	10 (5, 20)	9 (4.25, 15)	14 (10, 27.5)	0.071
Cough, n (%)	15 (24)	12 (26)	3 (20)	1
Chest pain, n (%)	1 (2)	1 (2)	0 (0)	1
Abdominal pain, n (%)	6 (10)	5 (11)	1 (7)	1
Arthralgia/arthritis, n (%)	6 (10)	3 (6)	3 (20)	0.146
Headache, n (%)	6 (10)	3 (6)	3 (20)	0.146
Myalgia, n (%)	4 (6)	4 (9)	0 (0)	0.564
Arthralgia/arthritis, n (%)	32 (52)	24 (51)	8 (53)	1
Joint involvement, n (%)				0.871
Single joint	20 (32)	16 (34)	4 (27)	
Multiple joints	18 (29)	13 (28)	5 (33)	
NO	24 (39)	18 (38)	6 (40)	
Fatigue, n (%)	11 (18)	6 (13)	5 (33)	0.115
Urinary involvement, n (%)	1 (2)	1 (2)	0 (0)	1
Hematologic abnormalities, n (%)	5 (8)	2 (4)	3 (20)	0.086
Seizures, n (%)	2 (3)	1 (2)	1 (7)	0.428
Limb paralysis, n (%)	0(0)	0(0)	0(0)	1
Night sweats, n (%)	20 (32)	13 (28)	7 (47)	0.211
Weight loss, n (%)	2 (3)	2 (4)	0 (0)	1
Signs				
Rash, n (%)	9 (15)	5 (11)	4 (27)	0.201
Lymphadenopathy, n (%)	13 (21)	9 (19)	4 (27)	0.716
Hepatomegaly, n (%)	15 (24)	12 (26)	3 (20)	1
Splenomegaly, n (%)	22 (35)	18 (38)	4 (27)	0.61
Joint swelling, n (%)	19 (31)	13 (28)	6 (40)	0.521
Neurological signs, n (%)	3 (5)	1 (2)	2 (13)	0.143
Laboratory Tests				
WBC (×10^9/L), Median (Q1, Q3)	6.95 (5.27, 8.44)	6.88 (5.22, 8.36)	7.15 (5.61, 8.58)	0.565
Hemoglobin (g/L), Mean ± SD	118.52 ± 14.34	119.7 ± 14.11	114.8 ± 14.9	0.273
Platelets (×10^9/L), Mean ± SD	229.39 ± 90.92	223.34 ± 92.44	248.33 ± 86.19	0.346
Neutrophil percentage (%), Median (Q1, Q3)	0.4 (0.28, 0.48)	0.38 (0.27, 0.48)	0.41 (0.37, 0.5)	0.393
Neutrophil count (×10^9/L), Median (Q1, Q3)	2.7 (1.96, 3.42)	2.52 (1.98, 3.24)	3.06 (2.09, 3.78)	0.206
Lymphocyte percentage (%), Median (Q1, Q3)	0.54 (0.46, 0.65)	0.55 (0.46, 0.65)	0.53 (0.43, 0.56)	0.421
Lymphocyte count (×10^9/L), Median (Q1,Q3)	3.66 (2.36, 4.91)	3.69 (2.37, 5.07)	3.64 (2.4, 4.62)	0.657
CRP (mg/L), Median (Q1, Q3)	6.32 (2.28, 17.01)	6.62 (2.85, 17.62)	2.76 (1.29, 9.21)	0.201
PCT (ng/mL), Median (Q1, Q3)	0.25 (0.25, 0.44)	0.25 (0.25, 0.44)	0.25 (0.25, 0.4)	0.495
IL-6 (pg/mL), Median (Q1, Q3)	13.92 (7.56, 22.18)	11.61 (7.03, 22.18)	16.74 (10.9, 21.95)	0.56
Ferritin (μg/L), Median (Q1, Q3)	148.8 (110.82, 235.45)	149 (110, 256.3)	133.7 (114.25, 193.85)	0.663
ESR (mm/h), Median (Q1, Q3)	23 (13, 32.5)	19.5 (10.5, 28.25)	36 (24.5, 45)	0.007
IgA (g/L), Median (Q1, Q3)	0.93 (0.56, 1.55)	0.86 (0.47, 1.49)	1.16 (0.72, 1.69)	0.187
IgG (g/L), Median (Q1, Q3)	10.31 (7.46, 12.45)	10 (7.36, 11.4)	10.99 (7.51, 15.8)	0.398
IgM (g/L), Median (Q1, Q3)	1.17 (0.8, 1.46)	0.98 (0.73, 1.35)	1.43 (1.18, 1.74)	0.011
ALT (U/L), Median (Q1, Q3)	42 (26, 72)	52 (27.25, 80.75)	28 (23, 39)	0.089
AST (U/L), Median (Q1, Q3)	55 (40, 93)	61 (39.58, 108.5)	45 (40.5, 57.5)	0.106
Albumin (g/L), Mean ± SD	37.6 ± 3.84	37.32 ± 3.78	38.46 ± 4.05	0.348
LDH (U/L), Median (Q1, Q3)	344 (276, 473)	356 (284.12, 484.25)	312.7 (272.5, 424.6)	0.481
Direct bilirubin (μmol/L), Median (Q1, Q3)	2.6 (2.3, 3.6)	2.6 (2.3, 3.6)	2.8 (2.2, 3.25)	0.993
Indirect bilirubin (μmol/L), Median (Q1, Q3)	4.9 (3.9, 6.5)	5 (3.92, 6.7)	4.9 (3.95, 5.88)	0.663
Creatinine (μmol/L), Median (Q1, Q3)	24 (19, 33)	25 (19, 33.75)	23 (18.3, 31.5)	0.663
CK (U/L), Median (Q1, Q3)	44 (31, 59.25)	44 (31, 59)	44 (32, 59.1)	0.959
CK-MB (U/L), Median (Q1, Q3)	18 (14, 22)	17 (14, 22)	19 (16, 22)	0.412
CD3 + T cells (%), Median (Q1, Q3)	78.44 (72.6, 82.02)	78.22 (71.5, 81.69)	78.86 (74.18, 82.65)	0.466
CD4 + T cells (%), Mean ± SD	30.21 ± 7.21	29.47 ± 7.16	32.29 ± 7.22	0.259
CD8 + T cells (%), Median (Q1, Q3)	33.2 (27.16, 37.77)	33.87 (27.16, 38.81)	33.08 (30.24, 35.61)	0.652
CD19 + B cells (%), Median (Q1, Q3)	10.82 (7.64, 18.86)	10.64 (6.47, 18.86)	11.6 (9.55, 16.74)	0.63
CD16/56 + NK cells (%), Median (Q1, Q3)	7.46 (5.86, 10.08)	7.46 (6.08, 11.48)	7.31 (5.17, 9.52)	0.421
CD4/CD8 ratio, Median (Q1, Q3)	0.89 (0.7, 1.05)	0.9 (0.69, 1.02)	0.86 (0.79, 1.34)	0.499
PT (s), Median (Q1, Q3)	13.5 (12.9, 14.05)	13.6 (13.05, 14.05)	13.27 (12.45, 13.9)	0.449
APTT (s), Mean ± SD	40.77 ± 7.85	41.96 ± 7.6	37.38 ± 7.89	0.097
Fibrinogen (g/L), Mean ± SD	3 ± 0.6	2.94 ± 0.61	3.15 ± 0.58	0.299
D-dimer (mg/L), Median (Q1, Q3)	1.03 (0.58, 1.99)	1.08 (0.6, 1.99)	0.74 (0.44, 2.9)	0.802
CSF WBC (×10^6/L), Median (Q1, Q3)	5 (3, 12.5)	4 (3, 5.75)	66 (14, 105.5)	< 0.001
CSF glucose (mmol/L), Mean ± SD	3.06 ± 0.58	3.12 ± 0.5	2.93 ± 0.71	0.366
CSF chloride (mmol/L), Mean ± SD	125 ± 3.55	125.61 ± 2.97	123.68 ± 4.4	0.152
CSF protein (g/L), Median (Q1, Q3)	0.11 (0.08, 0.14)	0.11 (0.07, 0.12)	0.13 (0.1, 0.2)	0.094
Blood culture positivity time (days), n (%)				0.513
*4*	17 (31)	13 (32)	4 (29)	
*5*	29 (54)	21 (52)	8 (57)	
*6*	4 (7)	4 (10)	0 (0)	
*7*	2 (4)	1 (2)	1 (7)	
*8*	2 (4)	1 (2)	1 (7)	
Complications				
Pneumonia, n (%)	14 (23)	11 (23)	3 (20)	1
Hip joint involvement, n (%)	6 (10)	3 (6)	3 (20)	0.146
Knee joint involvement, n (%)	4 (6)	4 (9)	0 (0)	0.564
Ankle joint involvement, n (%)	1 (2)	1 (2)	0 (0)	1
Femoral involvement, n (%)	4 (6)	4 (9)	0 (0)	0.564
Sacroiliac joint involvement, n (%)	2 (3)	0 (0)	2 (13)	0.056
Meningoencephalitis, n (%)	13 (21)	0 (0)	13 (87)	< 0.001
Treatment				
TMP-SMX + Rifampicin, n (%)	11 (18)	10 (21)	1 (7)	0.268
Doxycycline + Rifampicin, n (%)	14 (23)	7 (15)	7 (47)	0.028
Rifampicin + Ceftriaxone, n (%)	10 (16)	7 (15)	3 (20)	0.693
Doxycycline + Ceftriaxone, n (%)	4 (6)	3 (6)	1 (7)	1
TMP-SMX + Ceftriaxone, n (%)	2 (3)	2 (4)	0 (0)	1
TMP-SMX + Doxycycline + Rifampicin, n (%)	6 (10)	6 (13)	0 (0)	0.321
TMP-SMX+ Rifampicin + Ceftriaxone, n (%)	15 (24)	12 (26)	3 (20)	1
Outcome				
Relapse, n (%)	1 (2)	1(2)	0 (0)	1
Sequelae (arthritis), n (%)	1 (2)	0 (0)	1 (7)	0.242

Abbreviation: WBC (White Blood Cell Count), CRP (C-Reactive Protein), PCT (Procalcitonin), IL-6 (Interleukin-6), ESR (Erythrocyte Sedimentation Rate), IgA, IgG, IgM (Immunoglobulin A/G/M), ALT (Alanine Aminotransferase), AST (Aspartate Aminotransferase), LDH (Lactate Dehydrogenase), CK (Creatine Kinase), CK-MB (Creatine Kinase-Myocardial Band), PT (Prothrombin Time), APTT (Activated Partial Thromboplastin Time), CSF (Cerebrospinal Fluid), and TMP-SMX (Trimethoprim-Sulfamethoxazole).

### 3.2. Symptoms and signs

**Fig 3:**
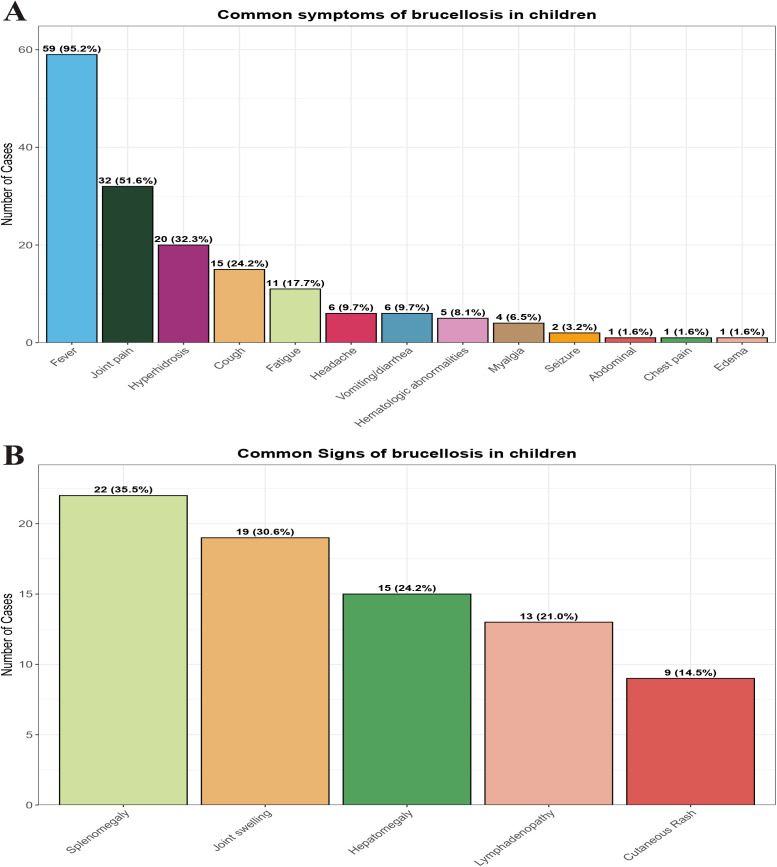
Common symptoms (A) and signs (B) of pediatric brucellosis in Yunnan Province, China, 2015–2024.

Fever was the most common symptom, observed in 59 cases (95.2%), followed by joint pain in 32 cases (51.6%). Hyperhidrosis and cough were reported in 20 cases (32.3%) and 15 cases (24.2%), respectively. Other symptoms such as fatigue, headache, and vomiting/diarrhea were less common (**Fig 3a**).

In terms of physical signs, splenomegaly was the most frequent, found in 22 cases (35.5%), followed by joint swelling in 19 cases (30.6%). Hepatomegaly and lymphadenopathy were observed in 15 cases (24.2%) and 13 cases (21.0%), respectively. Rash occurred in 9 cases (14.5%) (**Fig 3b).** There were no statistically significant differences in symptoms and signs between the groups (**Table 1).**

### 3.3. Laboratory tests

In laboratory tests, the erythrocyte sedimentation rate (ESR) showed a significant difference between the groups (p=0.007). The median ESR for the severe complication group was 36 mm/h, significantly higher than the 19.5 mm/h for the general group. Blood Immunoglobulin M(IgM) levels in the severe complication group were 1.43 g/L, compared to 0.98 g/L in the general group, with a statistically significant difference (p=0.011). Other laboratory markers showed no significant differences between the groups (**Table 1).**

**Fig 4:**
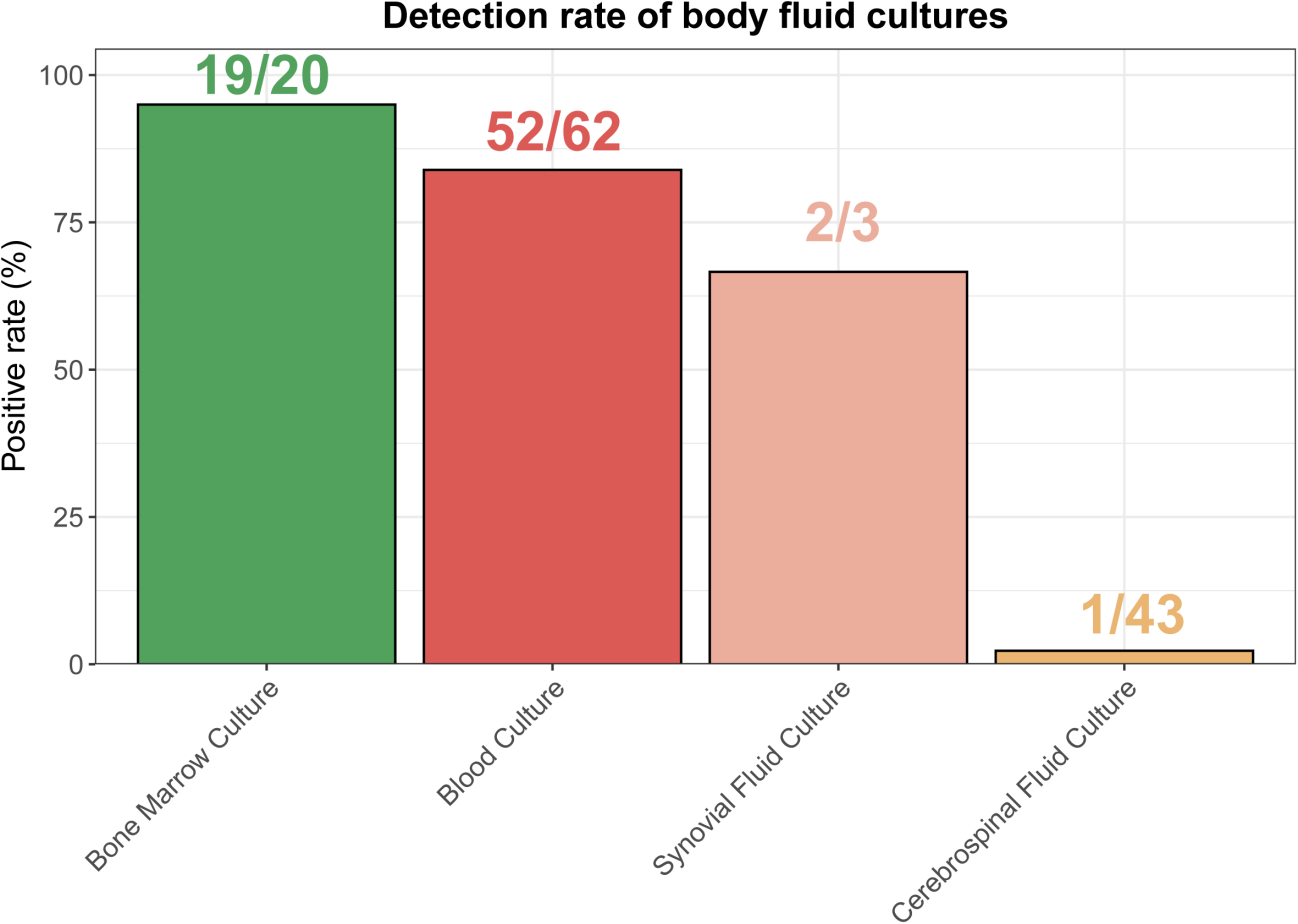
Positive differences among four types of body fluid cultures in pediatric brucellosis in Yunnan Province, China, 2015–2024.

Notably, there were significant differences in the positive culture rates of different bodily fluids. The positive rate of bone marrow culture was 95.0% (19/20), blood culture was 83.8% (52/62), synovial fluid culture was 66.7% (2/3), while cerebrospinal fluid culture had a much lower positive rate of 2.3% (1/43). (**Fig 4**).

### 3.4. Treatment

Among the 62 children included in the study, the most used treatment regimen was a combination of Cotrimoxazole, Rifampin, and Ceftriaxone (24.2%, 15/62). Other regimens included Doxycycline combined with Rifampin (22.6%, 14/62) and Cotrimoxazole with Rifampin (17.8%, 11/62). The combination of Rifampin and Ceftriaxone Sodium was used in 16.1% (10/62) of cases, while other combinations were less frequently used.

### 3.5. Outcomes

In terms of outcomes, 61 children did not experience relapse, with only 1 case (2%) of relapse. Additionally, one patient was left with a limp as a long-term sequela.

### 3.6. Construction of severe complications model for pediatric brucellosis

Among the 62 children with brucellosis, the following complications were observed: pneumonia (22,6%, 14 cases) was the most common, followed by meningoencephalitis (21.0%, 13 cases). In terms of joint involvement, the hip joint (9.7%, 6 cases) and sacroiliac joint (3.2%, 2 cases) were most frequently affected, while knee joints (6.5%, 4 cases), femur (6.5%, 4 cases), and ankle joints (1.6%, 1 case) were less affected. Meningoencephalitis and sacroiliac joint involvement were considered severe complications.

**Fig 5:**
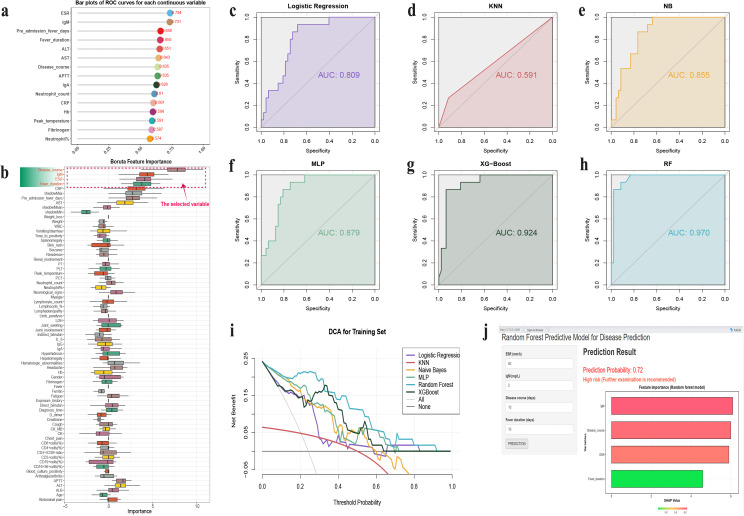
Severe pediatric brucellosis prediction model in Yunnan Province, China, 2015–2024. **(a)** ROC curve analysis, evaluating the AUC values of continuous variables to predict the risk of severe brucellosis. **(b)** Variable selection using the BORUTA algorithm to identify key variables associated with the prediction of severe disease, with the selected variables marked by the red dashed lines. **(c-h)** Comparison of AUC values for six prediction models (logistic regression, KNN, naive Bayes, MLP, XGBoost, and random forest), with the random forest model demonstrating the highest AUC value (AUC = 0.970), indicating optimal performance. **(i)** Decision curve analysis (DCA) to assess the net benefit of the six models at different threshold probabilities, with the random forest model showing the greatest net benefit. **(j)** A web-based calculator developed using ‘Shiny’ functions, designed to predict the risk of severe brucellosis based on clinical variables, providing real-time prediction results and the importance evaluation of each variable to assist clinical decision-making.

To assess the value of clinical variables in predicting severe complications of pediatric brucellosis, a multidimensional analysis using machine learning models was performed. First, univariate prediction for continuous variables was conducted (**Fig 5a**). ROC curve analysis revealed that ESR and IgM had AUC values of 0.734 and 0.731, respectively, suggesting limited predictive value. Next (**Fig 5b**), the ‘Boruta’ algorithm was used to select variables, and the disease duration, fever days, IgM, and ESR were identified as key predictive factors. Among the six machine learning models constructed, the Random Forest model exhibited superior predictive performance (AUC = 0.970), significantly outperforming XGBoost (0.924), MLP (0.879), Naive Bayes (0.855), Logistic Regression (0.809), and KNN (0.591) (**Fig 5c**–**5h**). The Random Forest model had an F1 score of 0.920, accuracy of 0.871, sensitivity of 0.979, and specificity of 0.533. DCA (**Fig 5i**) further confirmed that the Random Forest model provided the highest net benefit across all threshold probabilities. Based on these findings, a Shiny-based web calculator was developed (**Fig 5j**), integrating key predictive variables to provide clinicians with an immediate tool for severe risk assessment. These results demonstrated that combining inflammatory markers and machine learning approaches, particularly the Random Forest model, can effectively predict the risk of severe complications in pediatric brucellosis patients in non-endemic areas.

## 4. Discussion

This study presented the first systematic analysis of the clinical characteristics of brucellosis in children from non-endemic areas in Yunnan Province. The results revealed that the most common clinical manifestations were fever and joint pain. Serum markers, such as ESR and IgM levels, were significantly elevated in the severe complication cases. Meningoencephalitis emerged as the most frequent severe complication. Furthermore, the Boruta algorithm was employed to identify key predictive factors, leading to the development of a prediction model based on disease duration, fever duration, IgM, and ESR. The Random Forest algorithm demonstrated exceptional predictive performance. Additionally, the model has been translated into a web-based calculator tool, built on the Shiny framework, providing real-time risk assessment for clinicians.

Brucellosis is a zoonotic disease that impacts global human health, affecting multiple organs and systems throughout the body. Studies [14] predict that the incidence of brucellosis will gradually increase over the next few decades, potentially reaching its peak around 2,030. Recent research [15] indicates a rising trend in brucellosis cases in China, with the incidence increasing in southwestern non-endemic regions. Although Yunnan Province is not a traditional endemic area for brucellosis, the rising number of cases in recent years suggests that the potential risk to the pediatric population is increasingly being overlooked. This study observed a year-on-year increase in cases at the Kunming Children’s Hospital, particularly in 2023 and 2024, highlighting that brucellosis in non-endemic regions may not be receiving adequate attention and early diagnosis remains challenging. Additionally, the rising demand for beef, lamb, and dairy products in urban areas, coupled with increased cross-regional livestock trade, may be contributing to the rising number of cases.

In this study, the proportion of male patients was significantly higher (60%), which is related to the increased frequency of outdoor activities and greater opportunities for animal contact among male children. The age distribution showed a median age of 5 years, indicating that older children are more susceptible to brucellosis, which is consistent with previous reports of a higher incidence in children over 5 years old [16]. This is because older children are more likely to have direct contact with infectious sources, highlighting the need to focus on this age group. Notably, the seasonal distribution showed a peak in spring and summer, which aligns with the transmission patterns of brucellosis. Yunnan Province, located in a monsoon climate zone with distinct wet and dry seasons, experiences warm and rainy summers, which increase the risk of pathogen exposure during the livestock breeding season. This also correlates with the fact that children are more likely to engage in outdoor activities in the summer, increasing their chances of contacting with animals or poultry.

In this study, the most common clinical symptoms were fever (95.2%) and joint pain (51.6%), which are consistent with the main symptoms of brucellosis in other pediatric cases [16–18]. However, it is noteworthy that a small number of children in this study did not present with fever and lacked the typical “undulant fever” associated with brucellosis. Additionally, the affected joints in this study were primarily the hip and knee, which contrasts with the more commonly affected sacroiliac joints in adults [17,19]. Due to the incomplete blood-brain barrier in children, brucellosis is more likely to involve the central nervous system, and in this study, the proportion of children with meningoencephalitis exceeded 10%, while in adult cases, it typically does not exceed 5% [11,20]. This indicated that brucellosis in children often presents with atypical clinical manifestations, which can lead to misdiagnosis or missed diagnosis. Therefore, clinicians should be highly vigilant and make a comprehensive diagnosis based on the patient’s epidemiological history and laboratory tests to avoid neglecting the disease. We also observed that pneumonia was a relatively common complication in this cohort. This phenomenon may be associated with prolonged fever in children, combined with the fact that their respiratory system is structurally and functionally immature, rendering them more susceptible to secondary respiratory tract infections. In addition, Brucella infection itself may involve the respiratory system, manifesting with pneumonia-like clinical features, a tendency particularly evident in children with immature immune responses. Moreover, most patients in this study were from rural areas, where frequent contact with livestock and limited access to timely medical care may have further increased the risk of pulmonary involvement.

In terms of laboratory tests, this study found that bone marrow culture had the highest positive rate (95%), followed by blood culture (84%), while cerebrospinal fluid (CSF) culture had a low positive rate (2%). This suggested that bone marrow and blood cultures have higher sensitivity in diagnosing brucellosis, while CSF culture has limited diagnostic value. However, the positive rate of blood culture, a common diagnostic method, varies across different centers. One study [21], which analyzed 10,000 specimens, reported a blood culture positivity rate of 9.9%, bone marrow positivity at 10.4%, and fluid specimens like urine and milk had a positivity rate of up to 100%. A retrospective study [6] of 581 brucellosis patients also indicated a low blood culture detection rate (4.48%). Another study [22] involving 743 patients found that 370 (49.80%) had brucellosis bacteremia. However, blood culture positivity rates in the literature show significant heterogeneity, ranging from 4.48% to 78.75% [23–26]. This high blood culture positivity rate in our study can be explained by several factors. First, in our hospital, repeated blood cultures are routinely performed for suspected brucellosis cases, which increases the likelihood of detecting the pathogen. Second, the culture bottles used in our study contained nutrient-rich media, which are superior to standard broth and provide enhanced conditions for Brucella growth. These enrichment methods, together with the fact that many of our pediatric patients had prolonged household exposure to livestock (cattle and sheep) and presented with high fever, may contribute to the relatively high positivity rate observed in our cohort.Given the limitations of traditional culture methods, PCR technology and next-generation sequencing (NGS) are gradually becoming new trends in brucellosis diagnosis. PCR offers rapid, specific diagnostic results, while NGS can simultaneously detect multiple pathogens [27], further improving the accuracy and sensitivity of brucellosis diagnosis. In the future, molecular biology-based diagnostic methods may become essential tools for the rapid diagnosis of brucellosis.

In the development of the predictive model, this study used the Random Forest algorithm and identified factors such as disease duration, fever days, IgM levels, and ESR that are closely related to the occurrence of severe complications in brucellosis. These factors may influence the clinical manifestations and prognosis of the disease by modulating the body’s immune and inflammatory responses. Inflammatory markers such as ESR and IgM play an important role in the diagnosis and prognosis assessment of brucellosis [27,28]. Additionally, we have implemented a web-based tool to facilitate clinical risk assessment, enabling clinicians to input patient data in a calculator-like format for real-time prediction of severe complications.

However, this study also has certain limitations. First, it is a single-center study with a relatively small sample size, and since Yunnan Province is geographically vast, the findings from Kunming may not fully represent the epidemic situation across the entire province. Second, as a retrospective study, it inevitably carries the risk of selection bias. Third, although the blood culture positivity rate in this study was unusually high compared with previous reports, this may be attributed to our laboratory’s routine use of repeated cultures and enrichment broth methods, which enhanced the sensitivity of Brucella isolation. Fourth, IgM antibody levels were measured using a semi-quantitative ELISA kit, and results were interpreted according to the manufacturer’s designated cut-off values; this platform-specific approach may limit comparability across studies. Fifth, although machine learning techniques were employed to predict severe complications, the sample size was limited and external validation remains necessary to further assess the predictive accuracy of the model and to substantiate the findings of this study.

## 5. Conclusion

This study indicated that pediatric brucellosis should not be neglected in non-endemic areas like Yunnan Province, China. Our findings highlight that fever, disease course, and elevated inflammatory markers such as ESR and IgM are strongly associated with severe complications. Combining inflammatory markers with Random Forest models can effectively predict the risk of severe complications in pediatric brucellosis. However, due to resource limitations and lack of infrastructure during the study period, advanced diagnostic techniques such as PCR and antibiotic susceptibility testing were not systematically performed, representing an important limitation. Future studies with larger, multi-center cohorts and the integration of molecular methods are warranted to further validate our results and improve clinical decision-making.

## Supporting information

S1 Data The original data used in this study for analysis. (XLSX)
